# Personality traits explain the relationship between psychedelic use and less depression in a comparative study

**DOI:** 10.1038/s41598-024-60890-1

**Published:** 2024-05-03

**Authors:** David K. Sjöström, Emma Claesdotter-Knutsson, Petri J. Kajonius

**Affiliations:** 1 Child and Adolescent Psychiatry, Department of Clinical Sciences Lund, Faculty of Medicine, Lund University, Lund, Sweden; 2 Region Skåne, Child and Adolescent Psychiatry, Regional Outpatient Care, Lund University Hospital, Lund, Sweden; 3 Department of Psychology, Lund University, Lund, Sweden

**Keywords:** Mental health, Psychedelics, Drug use, Personality traits, Human behaviour, Epidemiology

## Abstract

Interest in psychedelics is increasing due to the potential for improved mental health and quality of life. However, adverse effects on mental health are still a concern. Personality traits have been suggested to both influence the psychedelic experience and mental health, and even be changed by psychedelic use. The present study describes for the first time a national sample of Swedish psychedelic users (n = 400) compared to a sex and age-matched control-group of non-users (n = 400) regarding mental health variables (depression, insomnia, problematic alcohol and drug use, and dissociation) and personality (Big Five). Data was collected in an online survey including individuals from 16 years of age who had at least one psychedelic experience. The main results reported psychedelic users as less depressed (Patient Health Questionnaire-9; PHQ-9) (d = − 0.29) and having more use of drugs (Drug Use Disorders Identification Test; DUDIT) (d = 1.27). In the Big Five personality traits, openness differed notably (d = 1.72), and the between-group effects in PHQ-9 were explained by lower neuroticism. Our findings reveal that psychedelic users report less depression and higher drug use, and this is partly due to personality traits. These results have implications on how we view psychedelic users and the use of psychedelic drugs.

## Introduction

There has been a resurgence of interest in how psychedelics influence mental health^1^. Classical psychedelics are psychoactive agents such as psilocybin (magic mushrooms), mescaline (peyote), lysergic acid diethylamide (LSD), ibogaine, 5-methoxy-N,N-dimethyltryptamine (5-MEO-DMT) and N,N-Dimethyltryptamine (DMT or N,N-DMT) which share their primary effect as 5-HT2A-receptor agonists^2^. The intensity and quality of the psychedelic experience induced by these substances in consciousness seems to predict positive mental health effects, such as less depression^3,4^. There are also reports of changes in personality traits^5,6^. In one clinical study with psilocybin changes in openness were sustained at one year follow-up^7^. For the first time, the present study analyzes data on mental health and personality traits in Swedish psychedelic users compared to a matched non-user control group.

Clinical interventions, such as psychedelic assisted therapy, are overall physiologically and psychologically safe to administer according to systematic reviews with close to no lasting adverse events to date^8–10^. It's worth noting that clinical trials exclude populations with risks such as psychosis and bipolar disorders and cardiovascular disease. Two recent systematic reviews report on psychedelic therapy for depressive symptoms and anxiety demonstrating significant effects in the short term but inconclusive at follow-up^11,12^, and reduce negative drug use behavior in substance use disorders^13,14^. Alongside reducing psychiatric symptoms, psychedelics are linked with sustained increase in well-being^15^.

As interest in psychedelic research unfolds, there are signs of an increased interest in recreational use of psychedelics^16,17^. Recreational, or naturalistic, use of psychedelics is associated with beneficial results like those of clinical trials regarding mental health^18^ and the risk of negative medical, psychological, and functional outcomes are considered low but still largely unknown^19^. There is a concern about psychedelic induced dissociation/depersonalization worsening long term pathology in vulnerable groups, although this relationship has not been established^20^. Furthermore, current psychedelic clinical trials demonstrate effects in reducing burden of substance use disorders while the picture for recreational use is a bit more unclear. A recent population survey in the US (N = 56,276) analyzed associations between lifetime use of recreational classic psychedelic use (LSD, psilocybin, and mescaline) and mental health^21^. The results were mixed, with LSD and psilocybin associated with higher risk of substance dependence and abuse, and mescaline linked with lower risk of substance use disorder, compared to non-users. In the same way, as clinical and naturalistic studies of psychedelics are associated with lower rates of depression symptoms, a recent cross sectional survey study showed contradicting associations between past-life use of psilocybin and higher risk of depression episodes in adolescents^22^.

In a safe and well-prepared context including professional preparation, dosing session psychological support and integration^23,24^ an interplay of individual factors such as the quality of the psychedelic experience and personality traits such as openness predict both short- and long-term effects^3,25^. Personality can be seen as a baseline of individual traits consisting of regularities in thoughts, feelings, and behaviors across time and situations, and is regarded as stable after reaching adulthood^26^. The most widely used measurement of personality is the Big Five model: neuroticism (vs. stability), extraversion (vs. introversion), openness (vs. conventionality), agreeableness (vs. antagonism), and conscientiousness (vs. disinhibition)^27^. Even though these personality traits are mostly stable throughout life, certain intense life events can still give rise to small effects of change in personality^28^, including psychedelic experiences^5,6^. The studies report mainly the personality domain openness to increase after psychedelic use, together with decrease in neuroticism. How psychedelics change personality is mostly unclear and how durable such effects might be is debatable^29^.

This present study compares mental health and Big Five personality traits between recreational psychedelic users and non-user controls, assessing mental health differences in terms of depression, insomnia, substance use, dissociation. In addition, we test whether differences in personality traits may explain mental health differences.

## Method

### Participants and procedure

Data was collected as part of an online survey of Swedish psychedelic users. The survey was collected by a professional survey institute and published in February 2023. An online link to the survey was advertised in online forums in Sweden interested in psychedelic research. The inclusion criteria for participation in the study was last-year use of classical psychedelics, at least 16 years of age, and native fluency in Swedish. The study did not impose any exclusion criteria. For two weeks, a sample of 400 psychedelic users were collected. This sample size was power calculated in the power calculator module in Jamovi version 2.3.28 to detect small group differences at Cohen’s d = 0.20 in the assessment of mental health^30^ and individual differences in personality^31^.

A control-group of 400 sex- and age-matched Swedish speakers with no experience of psychedelic use were recruited to answer the same online mental health survey excluding the psychedelic-specific questions. The controls were recruited by the same online survey company Ipsos and were matched from a survey-panel based on the sex and age demographics of the psychedelic users. The participants in the panel were contacted by phone numbers to participate via the Swedish state personal address register (SPAR) by Ipsos. None of the participants in the groups were paid to answer the survey or included in any other study.The study was approved by the Swedish ethical review authority (Dr: 2022-06810-02). All participants had to click a box on the first page of the survey, declaring to have read the study information and agreeing to the informed consent. All collection and analysis of the data was performed in accordance with regulations, guidelines, and ethical approval. Informed consent was obtained from all the participants for study participation.

### The current paper is part of a larger project on psychedelic users in Sweden. Instruments

All questionnaires are existing instruments that have been translated into Swedish and validated for both adolescent and adult populations. The instruments underwent a process of back-translation repeatedly until the integrity of the content was fully preserved.

*Patient Health Questionnaire (PHQ-9)* The PHQ-9 is a self-report measure used for diagnosing, monitoring, and measuring severity of depression^32^. It comprises 9 items, which correspond to the diagnostic criteria for major depressive disorder in the DSM-IV. Responses are based on a 0–3 scale, where respondents indicate the frequency of each symptom in the last two weeks. The PHQ-9 exhibited good internal consistency with Cronbach's alpha in the present study (α = 0.89). The PHQ-9 severity scale categorizes depressive symptoms into five levels: no symptoms (0–4), mild (5–9), moderate (10–14), moderately severe (15–19), and severe (20–27).

*Insomnia Severity Index (ISI)* The ISI measures the nature, severity, and impact of insomnia^33^. It is a 7-item self-report questionnaire assessing severity of sleep onset, sleep maintenance, and early morning awakening problems, sleep dissatisfaction, interference of sleep difficulties with daytime functioning, noticeability of sleep problems by others, and distress caused by the sleep difficulties. ISI demonstrated good internal consistency (α = 0.87). ISI uses a total score to indicate: no significant insomnia (0–7), subthreshold insomnia (8–14), moderate clinical insomnia (15–21), and severe clinical insomnia (22–28).

*Alcohol Use Disorder Identification Tool (AUDIT)* The AUDIT is designed to identify individuals with hazardous and harmful patterns of alcohol consumption^34^. It is a 10-item screening tool developed by the World Health Organization. The AUDIT displayed good internal consistency (α = 0.85). The AUDIT score for alcohol consumption ranges from low risk (0–7 points) to medium risk (8–15), high risk (16–19), and likely addiction (20–40).

*Drug Use Disorder Identification Tool (DUDIT)* The DUDIT identifies individuals with problematic drug use or drug dependence^35^. It consists of 11 items with a mixture of consumption patterns and drug-related problems. The DUDIT showed good internal consistency (α = 0.89). The DUDIT scoring ranges from 0 to 44, indicating increasing drug dependence with higher scores. Recommended cut-offs are 6 for males and 2 for females in the general population.

*Adolescent Dissociative Experience Scale (A-DES).* The A-DES measures dissociative experiences and has a language adapted for adolescents and young adults^36^. We used a shortened version of A-DES containing 20 items to reduce the number of items in the survey. The items asked respondents to rate how often particular dissociative experiences occur on a scale from 0 to 10. The A-DES reported high internal consistency (α = 0.93). A score over 80 indicates pathological manifestation of dissociation.

*Big Five personality* We used 30 items from the International Personality Inventory Pool (IPIP-NEO-30) to measure the Big Five traits Neuroticism, Extraversion, Openness, Agreeableness, and Conscientiousness^37^. This brief IPIP-NEO-30 showed good internal consistencies (N (α = 0.88), E (α = 0.81), O (α = 0.78), A (α = 0.75), and C (α = 0.78).

*Demographics* Participants were asked to self-report regarding gender (male or female), occupation (student, unemployed, or employed) and relationship status (single or in a partnership). Age was measured by self-report of current age (range 16–65). Socioeconomic status (SES) during their upbringing, as well as at the current moment, was self-assessed using a scale ranging from 1 to 10, allowing participants to compare themselves to others in society, with 1 representing the lowest and 10 representing the highest level of social and financial resources available.

### Statistical analysis

There were no suspicious response-styles in terms of repetitive, erratic, or chunks of missing values detected, indicating good input-data. We compared the group of psychedelic users and the non-user group regarding demographics, mental health (PHQ-9, ISI, AUDIT, DUDIT and A-DES), and personality (IPIP-NEO-30). To analyze differences between the groups we used independent t-tests and report effect sizes in standardized mean differences (Cohen’s d). If homogeneity of variance by further Levene’s test was significant then the more conservative ordinal t-test was chosen. According to recommendations in individual differences’ research, d < 0.10 would be considered trivial, and d > 0.30 of relevant interest^31^. The cut-off value for significance testing was set to *p* < 0.01. We furthermore used analysis of covariance (ANCOVA) which is a statistical technique that integrates group comparisons with linear regression. With ANCOVA, we were able to conclude group differences while adjusting for the effects of personality traits as covariates. In analyzing mental health differences between psychedelic users and non-users, the idea is that there might be underlying personality traits that relate both to psychedelic use as well as mental health. For instance, individuals with high openness or low neuroticism might be more likely to try psychedelics and might also be prone to good mental health. In conclusion, we supply a Pearsons’s correlation matrix for all study variables in supplemental materials. We used the open source Jamovi version 2.3.28 solid version for our statistical analysis.

## Results

The psychedelic-user group reported an average number of psychedelic experiences in their lifetime as M = 6.9 (Md = 7.0; SD = 4.4). The average age of the participants was 34.5 (SD = 8.5), range 16–63. Some 68% of the sample were male and 32% female. The average time spent on the questionnaire was M = 22 min (SD = 8).

Table 1 shows the comparisons of mean scores and standard deviations regarding mental health, personality traits, and demographics between psychedelic users (Group A) and non-users (Group B). In the mental health screening self-questionnaires significant effect sizes were low to moderate, such as the psychedelic users scored significantly lower in the PHQ-9 (d = − 0.29) and slightly lower in the ISI, indicating lower levels of symptoms related to depression and insomnia. The psychedelic users also scored notably higher on the DUDIT questionnaire (d = 1.27) indicating more drug use. The other screening tools, problematic alcohol use (AUDIT) and dissociation (A-DES) demonstrated no significant effects between groups. Correlational matrix and density plots for all study variables can be found in the supplements.

**Table 1:** The differences in mental Health, personality, and demographics between (A) psychedelic users (n = 400) and (B) matched non-users (n = 400).

	Group A		Group B			
Mental health	M	SD	M	SD	*p*	(d)
PHQ-9	4.9	4.9	6.5	6.1	< .01	− 0.29
ISI	13.8	5.5	14.8	5.3	.01	− 0.18
AUDIT	15.2	4.4	14.9	4.5	.34	0.07
DUDIT	16.6	5.2	11.4	2.7	< .01	1.27
A-DES	31.7	17.9	30.9	20.8	.53	0.04
Personality						
Neuroticism	2.4	0.9	2.6	0.9	.01	− 0.18
Extraversion	3.6	0.8	3.3	0.8	< .01	0.39
Openness	4.3	0.5	3.3	0.7	< .01	1.72
Agreeableness	4.3	0.5	3.8	0.6	< .01	0.88
Conscientiousness	3.7	0.7	3.7	0.7	.20	0.09
Demographics						
Gender (male)	68%		68%			
Employed	65%		78%			
Student	26%		12%			
Status (partner)	47%		61%			
Age	34.5	8.5	34.2	8.6	.56	0.04
SES Current (1–10)	7.1	1.7	6.4	1.7	.01	− 0.19
SES Upbringing (1–10)	5.6	1.9	6.0	1.9	< .01	0.42

PHQ-9 = Patient Health Questionnaire, range 0–27, ISI = Insomnia Severity Index, range 0–28, AUDIT = Alcohol Use Disorder Identification Tool, range 0–40, DUDIT = Drug Use Disorder Identification Tool, range 0–44, A-DES = Adolescent Dissociative Experience Scale, range 0–100. SES = Socioeconomic status, range 1–10. *p* = independent t-test significance. d = Cohen’s standardized mean difference.

Table 1 also shows differences in personality traits. Psychedelic users demonstrated significant differences in all Big Five traits compared to non-users, particularly in notably higher openness (d = 1.72) and agreeableness (d = 0.88). Furthermore, sex and age were similar between groups according to the matched study design. However, psychedelic users rated their current individual socioeconomic status (SES) higher (d = 0.42) while their family home upbringing SES as slightly lower.

Table 2 presents the two largest differences in mental health, depression (PHQ-9) and drug use (DUDIT), between Group A and B, when controlling for the Big Five personality traits. In Table 2, the analysis of covariance (ANCOVA) with PHQ-9 as dependent variable reported as insignificant (F = 3.7, p 0.05, η^2^ = 0.00), when controlling for the Big Five personality traits as covariates. The trait neuroticism explained the most (F(1,767) = 211.5, η^2^ = 0.23), and the explained PHQ-9 variance decreased more than 50% (from 2 to 0%). The results of the ANCOVA with DUDIT as dependent variable reported a decrease from 29 to 20% when controlling for the Big Five personality traits but remained significant (F(1, 767) = 186.1, *p* < 0.001).

**Table 2:** The difference in depression (PHQ-9) between (A) psychedelic users and (B) matched non-users, controlling for Big Five personality with ANCOVA.

	Depression (PHQ-9)			Drug use (DUDIT)		
	F	*p*	η^2^	F	*p*	η^2^
Group A vs B (without personality)	16.0	< .001	.02	303	< .001	.29
Group A vs B	3.7	.05	.00	186.1	< .001	.20
Neuroticism	211.5	< .001	.23	3.8	.05	.00
Extraversion	2.1	.15	.00	2.0	.16	.00
Openness	0.7	.41	.00	0.8	.39	.00
Agreeableness	1.7	.19	.00	18.0	< .001	.02
Conscientiousness	10.7	< .001	.01	5.4	.02	.01

F = Fischer’s ratio between-group versus within-group variance. *p* = significance test by ANCOVA. η^2^ = explained % variance of dependent variable.

## Discussion

The results show that Swedish psychedelic users report lower rates of depression compared to matched controls. The psychedelic users also demonstrated higher problematic drug use as measured by DUDIT. The psychedelic users did not report more dissociation symptoms nor problematic alcohol use compared to controls. This could add to the growing evidence of psychedelic use being associated with low harm potential.

Based on the antidepressant effects documented in several clinical trials^11^, it could be that the use of psychedelics explains the lower depression symptoms as seen in the present sample and other survey studies^18^. However, it could also be that psychedelic users differ in personality traits, leading to different susceptibilities to depression or drug use behaviors. Speculatively, such differences in personality traits could be due to certain personalities seeking out psychedelic use, or that psychedelic use changes personality traits. Personality traits have been shown to predict use and experiences of psychedelics^2^, as well as predicting mental health. Psychedelic users displayed close to two standard deviations higher openness (d = 1.72). This implies that almost 98% of the psychedelic users had higher openness than non-users. This is considered an unusually large effect in psychological research. Similarly, agreeableness and extraversion were higher and neuroticism lower with psychedelic users. Controlling for Big Five personality explained most of the differences in depression and one third of drug use between groups. To our knowledge, this is one of the first findings in psychedelic literature showing how baseline individual characteristics are important for understanding the use of psychedelics.

It could be seen as contradictory that psychedelic users report being similar or higher in mental health and SES compared to a matched population, while having notably higher rates of overall problematic drug use as measured by DUDIT. One explanation could be that the DUDIT is asking about illegal drug behavior disregarding the type of drug whether it is recreational cannabis, psychedelics, or dependence-inducing heavy drugs^19,35^. It can be speculated that the psychedelic users mostly referred to their use of psychedelics when answering DUDIT, which is not sensitive enough to separate drugs with different impact on mental health and SES. This would confound the negative connotation of drug use seeing there is a growing body of reported personal meaningful experiences from psychedelic use^37,38^. It is also of interest that the psychedelic users did not report more dissociative symptoms, since it is being a concern linked to hallucinogen use^20^. The risk of sustained dissociation regarding drug use is likely modulated by individual psychological vulnerabilities associated with neuroticism. This would be in line with harm reduction traditions in clinical trials and screening for family history of dissociation and other psychotic conditions.

Among the psychedelic users, we found a large effect in the Big Five personality trait openness. This finding is consistent with other studies^5,6^. The open-minded person is prone to novel behaviors and will seek out and try psychedelics more than others. Openness can also relate to mental health^39^. Openness has shown to be associated with the quality of acute effects^40^ and the degree of mystical experiences in a psychedelic experience. These in turn predict beneficial mental health outcomes^25^. In addition, studies also indicate that openness could be increased after a psychedelic experience, even as far as a year afterwards^5^. A large personality difference was also observed in agreeableness. Such personality differences could partly be due to selection bias of who voluntarily was interested and amicable enough to choose to invest time and effort into answering a survey on a psychedelic forum. This brings to the forefront the need for more personality measurements in survey designs and psychedelic research.

The observation that psychedelic users reported a significantly lower SES during upbringing compared to their current SES and that this dynamic was not seen in the non-psychedelic users, may reflect the SES development coupled to the underlying personality structure of psychedelic users, particularly in terms of high openness^41^. It could furthermore also be a consequence of the subjectiveness of self-assessing socio-economic status^42^.

A cross-sectional study design prohibits any causal conclusions and interpretations. In addition, participants volunteering to a survey bring a self-selection bias that needs to be acknowledged^43^. For instance, it could be that participants choosing to fill out a survey on psychedelics have a particular bias to the subject, or that they are particularly open-minded and agreeable. However, in a comparatively matched study design such as the present, such bias is likely for both groups being compared. The matching of participants was validated in having the same sex and age-characteristics; however, psychedelic users rated their individual socioeconomic status (SES) significantly higher than non-users. Relying on other research, this may be a result due to lesser depression, lower neuroticism, and higher openness characterizing persons with higher education and higher income^44^. Furthermore, it is plausible to speculate that differences in SES between the groups may be attributed to geographical factors, which was not directly measured in the survey study.

Another limitation was the use of short clinical screening instruments. However, internal consistencies were high, and the data was collected by a professional survey institute. Arguably, using short screening instruments was a reasonable compromise for the samples as well as for suiting the widespread age-groups (16–63). For instance, the adult Dissociation Experience scale (DES) and A-DES are both scales designed to measure dissociative experiences, but target different age groups: adults and adolescents, respectively. While covering similar dissociative domains and structured similarly with a Likert scale for responses, A-DES differ somewhat in language and item content to be simpler and generally reflect experiences. This specificity may reduce the A-DES's applicability and accuracy when measuring dissociation in adults, potentially overlooking adult-specific dissociative experiences and nuances. Consequently, this limitation should be considered when evaluating the suitability of the A-DES for adult populations in research contexts.The present study is one the first of its kind reporting that the mental health domain of depression, if anything is slightly lower among psychedelic users, but also that this in part may have to do with differences in personality traits. Particularly openness has shown to be higher among psychedelic users, which the present study also was able to confirm. We argue for including personality measurements in psychedelic research to be able to explore how individual differences of psychedelic users’ interplay with the psychedelic experience and with self-reports of mental health such as depression.

### Supplementary Information

Supplementary Information.

## Data Availability

All data is stored at the University of Lund and is available upon request by sending an email to the corresponding author.
