# Supplementary material for: Personality traits explain the relationship between psychedelic use and less depression in a comparative study
Source: Sci Rep. 2024 May 3;14:10195. doi: 10.1038/s41598-024-60890-1 (PMC11068892; doi:10.1038/s41598-024-60890-1)

Appendix

Table A1

Correlational Matrix of Study Variables

	PHQ-9	ISI	AUDIT	DUDIT	A-DES	N	E	O	A	C
PHQ-9	—									
ISI	**.63**	—								
Audit	.19	.17	—							
Dudit	.08	.07	**.35**	—						
A-des	**.42**	**.33**	.29	.30	—					
Neuroticism (N)	**.57**	**.42**	.13	.04	**.31**	—				
Extraversion (E)	**-.34**	-.21	.03	.04	-.16	**-.43**	—			
Openness (O)	-.11	.06	-.04	**.33**	.03	-.08	.26	—		
Agreeableness (A)	-.20	-.12	-.16	-.07	-.14	-.16	**.32**	**.47**	—	
Conscientiousness (C)	**-.33**	-.21	-.16	-.11	-.19	**-.38**	**.41**	.11	**.32**	—
Gender	-.02	-.01	-.20	.08	.02	.15	.00	.12	**.30**	.14
Age	-.05	.06	-.14	-.11	-.07	-.08	-.04	-.00	.14	-.01

Note. All correlations above Pearsons $r > .12$ are significant at $p < .01$. All correlations above .30 are in bold.

Figure A1

Density plots for study variables.

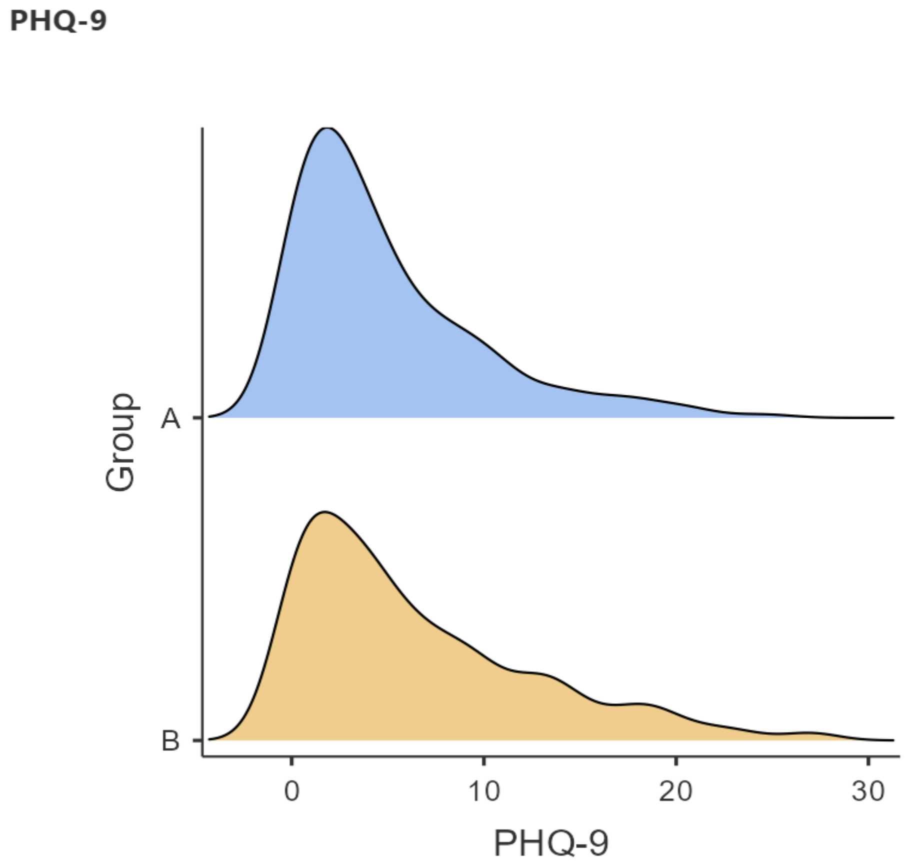

ISI

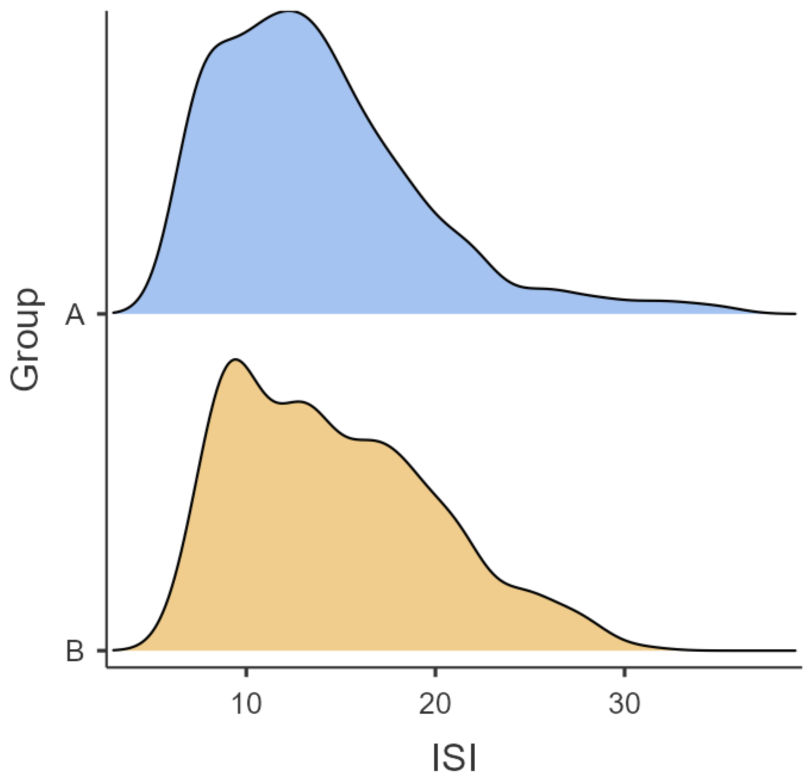

>

AUDIT

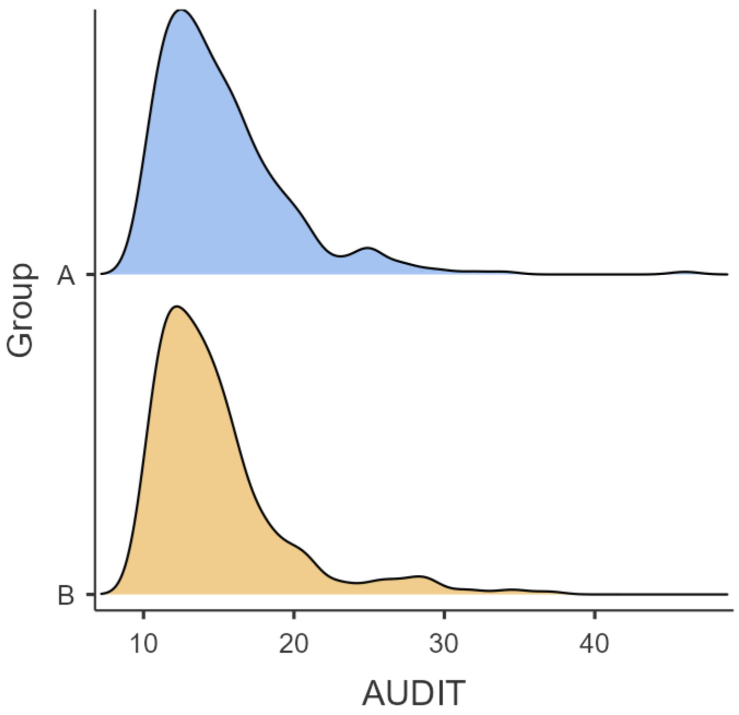

DUDIT

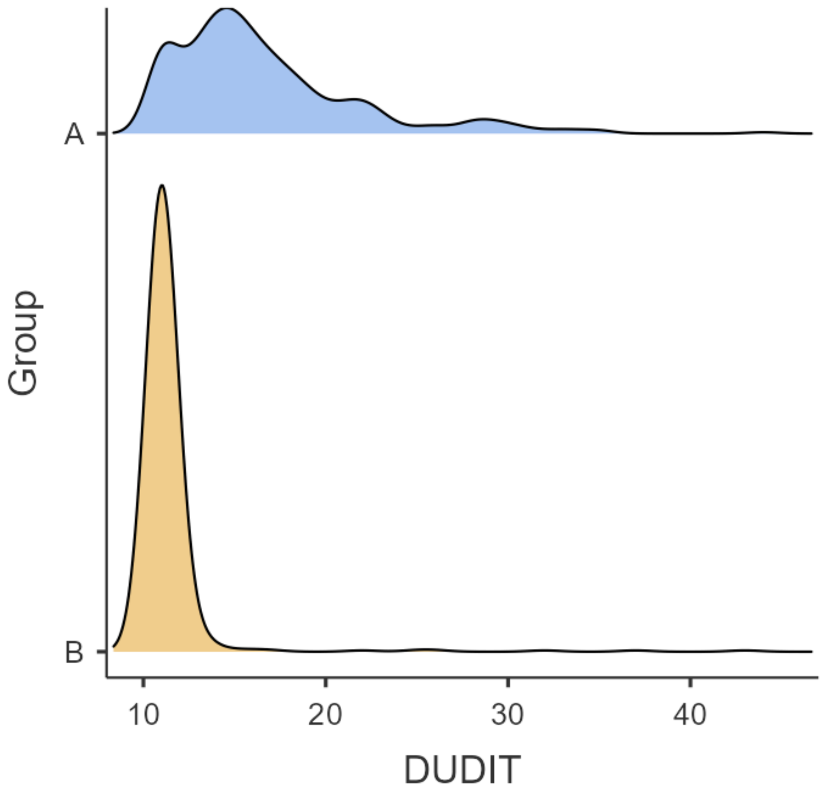

A-DES

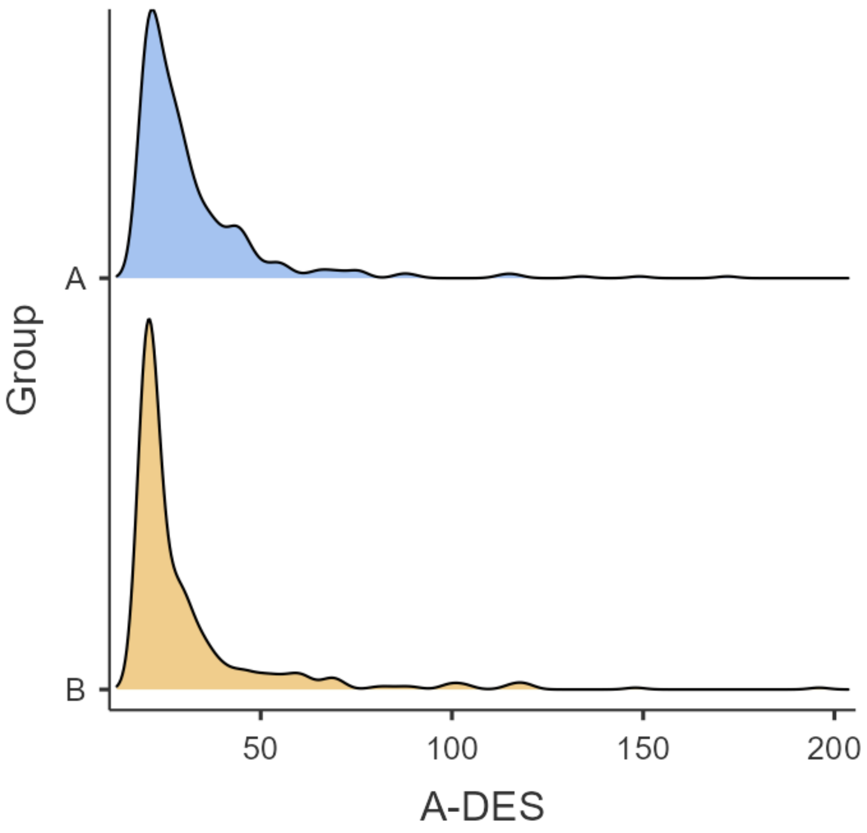

>

Neuroticism

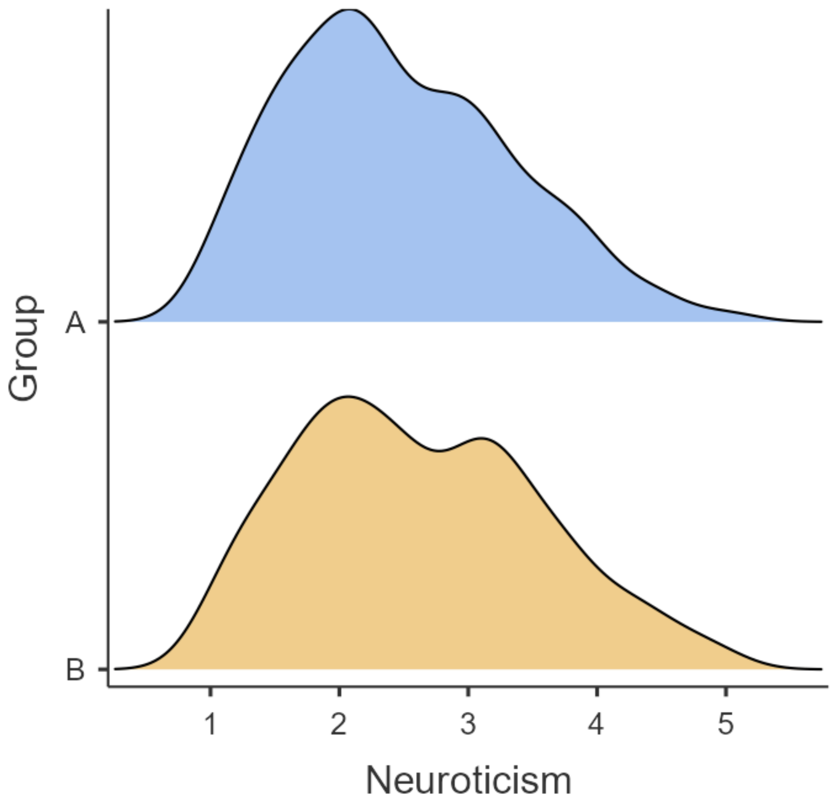

Extraversion

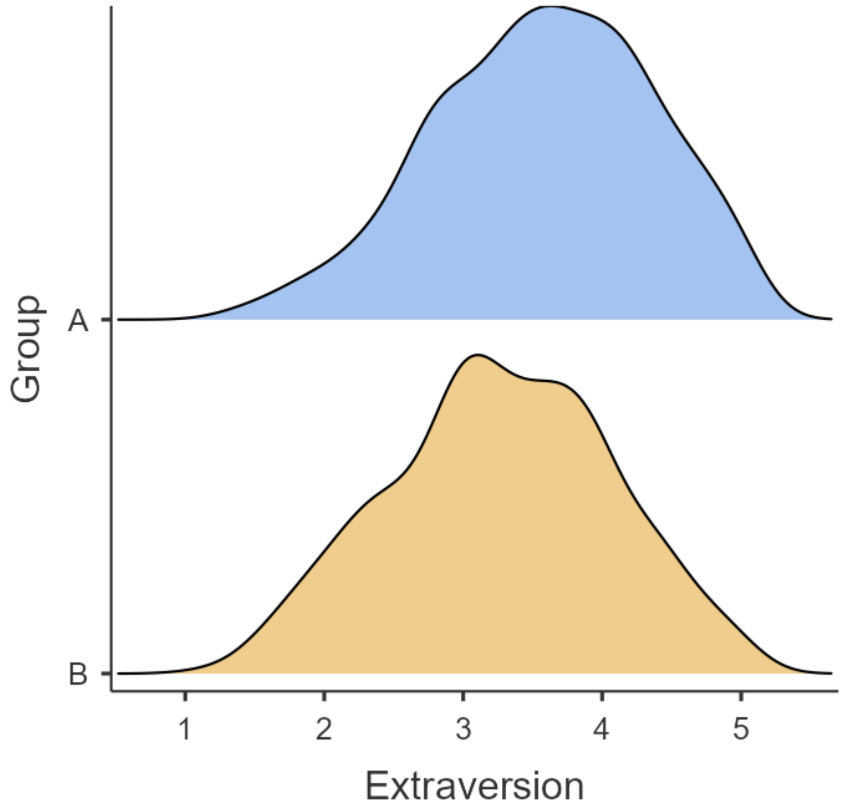

Openness

>

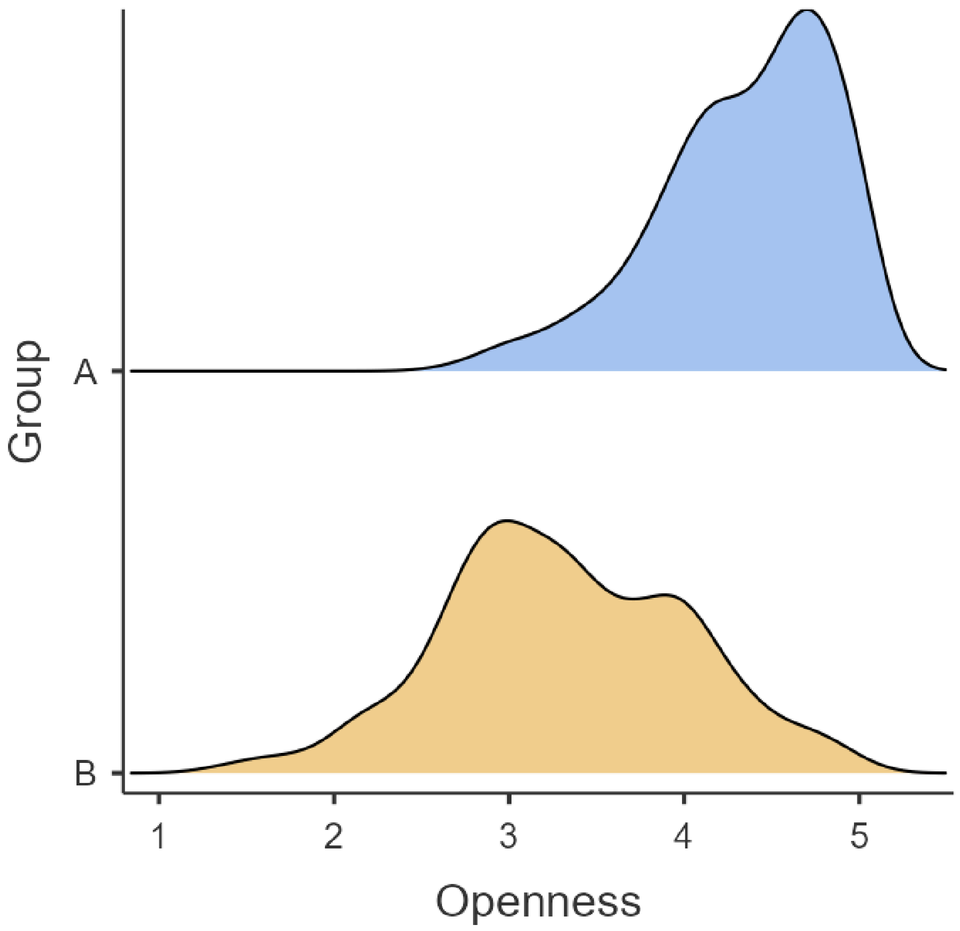

Agreeableness

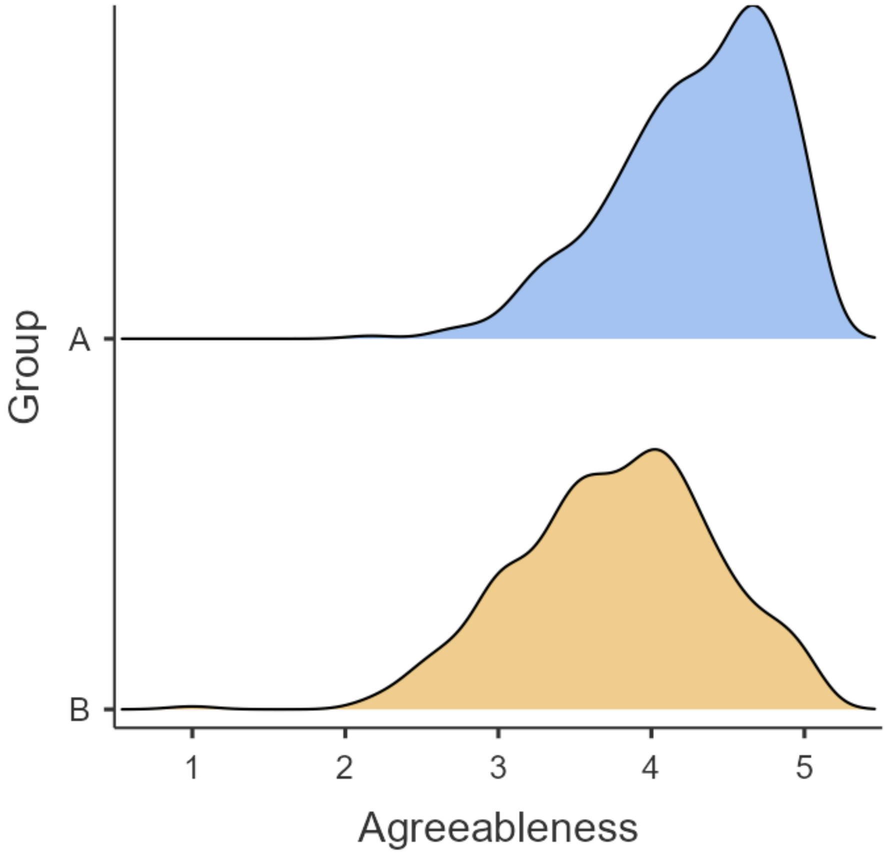

Conscientiousness

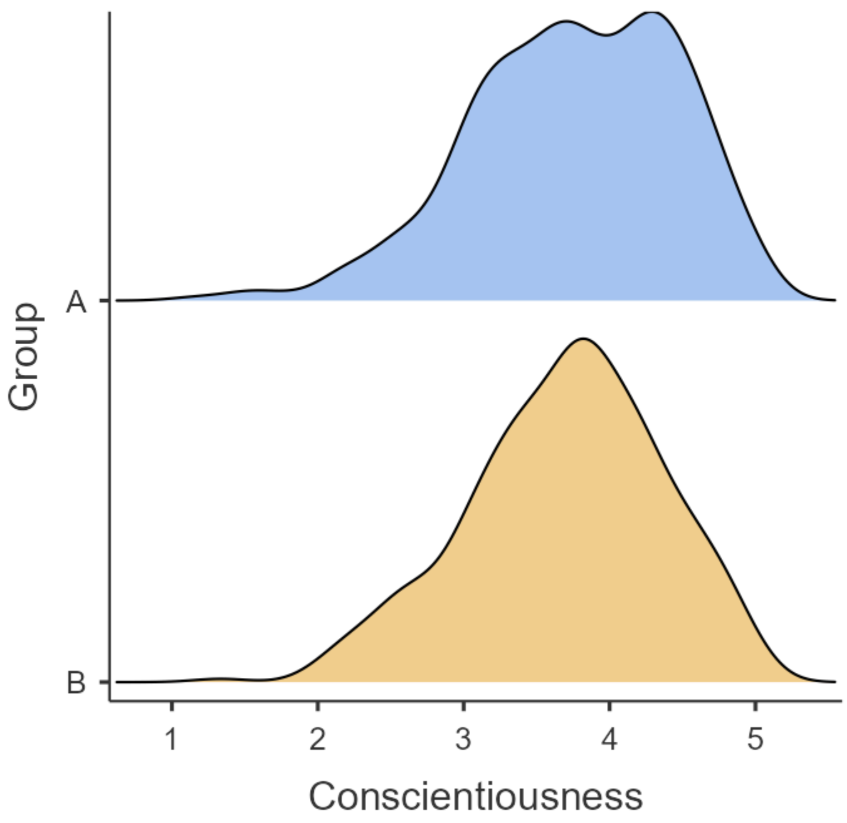

Supplement: Supplementary file 1 — Supplementary Information. [file 41598_2024_60890_MOESM1_ESM.pdf]
